# Cerebral Microbleeds in a Stroke Prevention Clinic

**DOI:** 10.3390/diagnostics10010018

**Published:** 2019-12-30

**Authors:** A-Hyun Cho, Lara Wadi, Daniel Chow, Peter Chang, David Floriolli, Krunal Shah, Annlia Paganini-Hill, Mark Fisher

**Affiliations:** 1 Department of Neurology, University of California, Irvine, Orange, CA 92868, USA; ahyun@catholic.ac.kr (A.-H.C.); wadil.efd@gmail.com (L.W.); krunals@hs.uci.edu (K.S.); apaganin@uci.edu (A.P.-H.); 2 Department of Neurology, Catholic University of Korea, College of Medicine, Seoul 06591, Korea; 3 Department of Radiological Sciences, University of California, Irvine, Orange, CA 92868, USA; chowd3@hs.uci.edu (D.C.); changp6@hs.uci.edu (P.C.); dfloriol@hs.uci.edu (D.F.); 4 Department of Pathology & Laboratory Medicine, University of California, Irvine, Orange, CA 92868, USA

**Keywords:** cerebral microbleeds, infarction, stroke prevention, cerebrovascular disease/stroke, CT, MRI

## Abstract

The objective of this study was to assess the effectiveness of a stroke clinic in stroke prevention and progression of cerebral microbleeds (CMB). We conducted a retrospective observational study of patients who visited a stroke clinic between January 2011 and March 2017. Susceptibility-weighted imaging (SWI) MRI studies were obtained at baseline and follow-up visits to identify new infarctions and CMB progression. Patients with CMB who also underwent brain computed tomography (CT) imaging were identified and their cerebral arterial calcification was quantified to evaluate the relationship between the extent of intracranial calcification and CMB burden. A total of 64 stroke patients (mean age 73.1 ± 11.0, 47% males) had CMB on baseline and follow-up MRI studies. During a mean follow-up period of 22.6 months, four strokes occurred (4/64, 6%; 3 ischemic, 1 hemorrhagic), producing mild neurological deficit. Progression of CMB was observed in 54% of patients with two MRIs and was significantly associated with length of follow-up. Subjects with intracranial calcification score > 300 cm^3^ had higher CMB count than those with scores <300 cm^3^ at both baseline (12.6 ± 11.7 vs. 4.9 ± 2.2, *p* = 0.02) and follow-up (14.1 ± 11.8 vs. 5.6 ± 2.4, *p* = 0.03) MRI evaluations. Patients with CMB had a relatively benign overall clinical course. The association between CMB burden and intracranial calcification warrants further study.

## 1. Introduction

Cerebral microbleeds (CMB) are MRI-demonstrable lesions corresponding to focal hemosiderin depositions that are indicative of underlying microhemorrhages [1]. These lesions persist with time, which allows the evaluation of cumulative microbleed burden. CMB detection is clinically relevant given that microbleeds may serve as a biomarker for increased risk of ischemic as well as hemorrhagic stroke [2]. CMB frequently coexist with ischemic stroke syndromes. This has been previously referred to as “mixed cerebrovascular disease”, a term which combines clinical and subclinical disease with both ischemic and hemorrhagic elements [3,4]. As a result, anticoagulation decisions and the need to prevent ischemic stroke, while not provoking hemorrhagic stroke or worsening CMB, can be challenging for the stroke clinician [4,5,6,7,8]. Accordingly, we hypothesized that the management of CMB patients by a stroke neurologist attentive to the complexities of mixed cerebrovascular disease would result in effective stroke prevention. In the current study, we investigated stroke events and the progression of CMB in patients who were initially evaluated and managed in a stroke clinic. We attempted to identify biomarkers for progression of CMB, using a recently proposed conceptual framework in which CMB development is associated with arterial abnormalities related to dysfunctional regulation of cerebral blood flow [9]. We therefore analyzed clinical outcomes and intracranial calcification in patients with CMB.

## 2. Results

### 2.1. Patients

Of the 348 patients who visited the stroke clinic at the UC Irvine Medical Center between January 2011 and March 2017, 66 (19%) patients had CMB on MRI; 64 of these had a follow-up visit and were included in this study. The mean age was 73.1 ± 11.0 years. Forty-seven percent were males. The ethnic distribution was 48% White, 34% Asian, 14% Hispanic, and 3% Black. The clinical diagnoses of the patients were ischemic stroke (*n* = 33, 52%), cerebral amyloid angiopathy (*n* = 11, 17%), vascular dementia (*n* = 10, 16%), intracerebral hemorrhage (*n* = 4, 6%), and other conditions (*n* = 6, 9%). The most common probable cause of CMB was hypertension alone (*n* = 31, 48%), followed by the combination of hypertension and chronic kidney disease (*n* = 11, 17%), hypertension and cerebral amyloid angiopathy (*n* = 9, 14%), and hypertension with chronic kidney disease and cerebral amyloid angiopathy (*n* = 1, 2%).

### 2.2. Stroke Outcome at Follow-Up

The 64 patients were followed for an average of 22.6 months. During this time, four clinical strokes occurred (3 ischemic strokes, 1 hemorrhagic stroke; 1.7% annualized ischemic stroke rate; 0.6% annualized hemorrhagic stroke rate). Three strokes were related to mild but permanent neurological deficit (1 facial weakness, 1 hemiparesis, 1 cognitive decline). All patients were treated with strict attention to blood pressure control and judicious use of antithrombotic medications.

### 2.3. CMB Progression on Follow-Up MRI Studies

**Figure 1:**
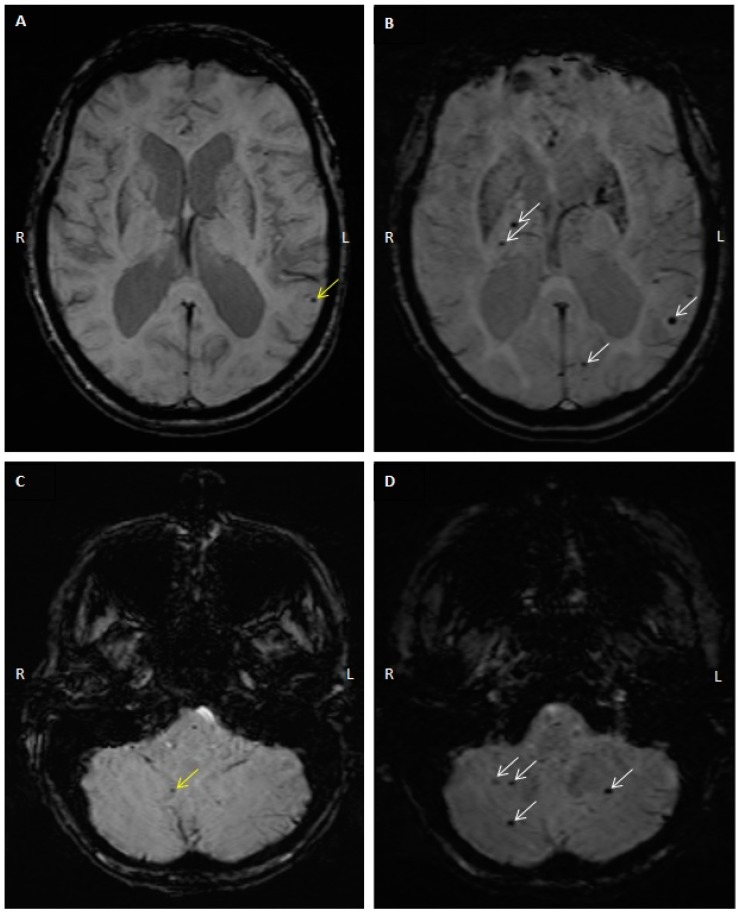
Baseline and follow-up axial susceptibility-weighted imaging showing CMB count progression. Axial SWI from the same individual showing cerebral microbleeds (CMB) in two sections at the initial visit shown in (**A**,**C**), and at the follow-up visit 36 months later shown in (**B**,**D**). (**A**) Left lobar microbleed (yellow arrow). (**B**) New right thalamic and left lobar microbleeds (white arrows). (**C**) Right cerebellar microbleed (yellow arrow). (**D**) New cerebellar microbleeds (white arrows).

Follow-up MRI was performed in 46 of the 64 patients. The mean interval between initial and follow-up MRI was 26.5 months (range: 1 to 77). Progression of CMB was observed in 25 patients (54%), with 14 having a CMB count increase of ≥3. Figure 1 is an example of baseline and follow-up axial susceptibility-weighted imaging (SWI) showing CMB count progression in one patient. Among the 46 patients who had follow-up MRIs, there were 26 males and 20 females; there was no observable association between sex and CMB progression in our study population (57% of males with no CMB progression as compared to 56% with CMB progression). Age, vascular risk factors, chronic kidney disease, cerebral amyloid angiopathy, and use of antithrombotics showed no association with CMB progression (Table 1). Mean MRI follow-up interval was significantly associated with CMB progression (18.7 (no progression) vs. 33.6 (progression) months, *p* = 0.003, Table 1). The majority of patients who had evidence of stroke between the initial and follow-up visits (3/4) had progression of their CMB count on follow-up MRI.

### 2.4. Intracranial Calcification

**Figure 2:**
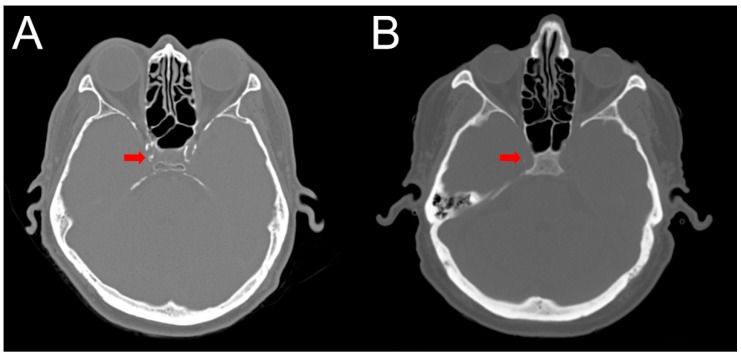
Axial non-contrast head computed tomography (CT) scans comparing patients with a high and low burden of calcification. Head CT obtained from a patient with a “high” burden of calcification (**A**) within the cavernous carotid (denoted by the arrow), which measured 3093 cm^3^. For comparison, axial non-contrast head CT of a patient with a “low” burden of calcification (**B**) within the cavernous carotid, which measured 106 cm^3^.

The assessment of calcification with computed tomography (CT) was performed in 26 patients. Calcification scores ranged from 32 to 4021 cm^3^ with a mean of 877. Patients with intermediate calcification scores (300–1000 cm^3^) had mean CMB counts similar to the patients with high calcification scores (>1000 cm^3^). Excluding one patient with many but not quantifiable CMB on both initial and follow-up, these two groups had higher CMB counts than those with scores <300 cm^3^ in both the initial (12.6 ± 11.7 vs. 4.9 ± 2.2, *p* = 0.02) and follow-up (14.1 ± 11.8 vs. 5.6 ± 2.4, *p* = 0.03) MRI studies (Table 2, Figure 2).

## 3. Discussion

We describe a cohort of 64 patients with CMB followed in a stroke clinic over an average period of two years. We demonstrate that (1) the overall clinical course of patients with underlying CMB was relatively benign, (2) a significant proportion of the 46 patients who had baseline and follow-up MRIs (54%) had progression in their CMB count at their follow-up MRI, (3) a higher CMB count at follow-up was significantly related to length of follow-up and not with demographic or stroke risk factors, and (4) a higher cerebral arterial calcification score was significantly associated with a higher CMB count.

Previous studies have shown that CMB tend to persist with time on MRI using high-field and thin-slice parameters in patients who were followed for up to nine years [10,11]. Here we have shown that not only do CMB persist after two years, but that new microbleeds occur frequently during that time frame, thus indicating the potential need to conduct follow-up MRI studies at shorter intervals. At follow-up, 6% (4/64) of the patients reported having had a clinical stroke with mild neurologic deficits. Out of these four patients, three had an increase in the number of their CMB on follow-up MRI. Although this finding was not statistically significant, it does suggest a vulnerability to stroke in patients with CMB progression detected on MRI. Indeed, the presence of CMB has been associated with an increased rate of ischemic and hemorrhagic strokes [2].

The literature suggests that the presence of four or more microbleeds may increase the risk of cognitive decline and dementia [12,13]. Most patients in this study did not have a worsening of their cognitive function or signs of neurologic impairment at their follow-up visit in the clinic, suggesting that the increase in CMB count in this patient population with pre-existing microbleeds was essentially subclinical. Indeed, the majority of patients with CMB at baseline had a benign overall clinical course during the study period. They were treated with close attention to tight control of their blood pressure, and this has been shown to reduce the risk of incident strokes and dementia in patients with hypertension [14].

We also showed that intracerebral arterial calcification is associated with CMB, with higher vascular calcification scores obtained in patients with a larger number of CMB. Some studies have reported more extensive vascular calcification in both intracranial and extracranial arteries (internal carotid and coronary arteries) in patients with cerebral small vessel disease, including those with CMB [15,16,17]. Vascular calcification could disrupt the ability of brain arterioles to compensate for changes in systemic blood pressure, leading to a rise in the intraluminal pressure within the microvasculature and subsequent erythrocyte extravasation contributing to microhemorrhage formation [9,18,19,20]. The potential mechanisms relating vascular calcification and CMBs are complex and further studies are needed to gain a better understanding.

This study has limitations. The observational retrospective design of the study precludes making causal associations between the presence of intracerebral arterial calcification and CMB. The follow-up time was longer in patients showing CMB progression (33 vs. 19 months); progression of CMB might develop with longer follow-up. In addition, a larger sample size is needed to draw more definitive conclusions about clinical outcomes such as stroke and cognitive function associated with CMB progression. Existing data in the literature suggests associations between CMB presence and cerebral amyloid angiopathy [21], hypertension [22], and chronic kidney disease [23]. In our study, we report the comparison of the progression of CMB in association with the various vascular risk factors. With only 21 and 25 subjects in the two groups (no CMB progression and CMB progression, respectively), we had low statistical power to detect significant differences even for hypertension, which showed the greatest difference in proportions (67% of subjects with no CMB progression had hypertension as compared to 88% of subjects with both hypertension and CMB progression).

## 4. Materials and Methods

### 4.1. Standard Protocol Approvals, Registrations, and Patient Consents

This study was approved by the Institutional Review Board at University of California, Irvine (approval code: 2017-3590, approval date: 17 May 2018). Requirements for patient consents were waived due to the retrospective design of the study.

### 4.2. Patient Selection

We identified patients who visited a stroke prevention clinic at the UC Irvine Medical Center between January 2011 and March 2017. Patients who had CMB on susceptibility-weighted imaging magnetic resonance imaging were included for this analysis. Medical records were reviewed to obtain information about age, sex, vascular risk factors (hypertension, smoking, hyperlipidemia, diabetes), medications (antithrombotic and cholesterol-lowering medications), and history of chronic kidney disease and coronary artery disease. We determined the likely underlying etiology of CMB in each patient, e.g., hypertension, cerebral amyloid angiopathy, chronic kidney disease, and secondary microbleeds (hemorrhagic infarction or microinfarction) [9,21]. The timing of the follow-up visit was determined by the attending stroke physician.

### 4.3. MRI and CMB Identification

Baseline and follow-up MRIs were studied at initial and follow-up clinic visits set by the stroke physician. MRI examinations were conducted using a 1.5-tesla or 3-tesla scanner and images were obtained using the susceptibility-weighted imaging sequence. For the 1.5T MRI scans, we used the following parameters: repetition = 49 ms, echo time = 40 ms, flip angle = 15 degrees. For the 3T scans, the parameters were: repetition time = 27 ms, echo time = 20 ms, flip angle = 15 degrees. The slice thickness was 2 mm and the interlayer spacing was 0 mm for both types of scans. Susceptibility-weighted imaging was individually examined by an experienced radiologist and by a neurologist who were both blinded to the participants’ clinical information. Definite CMB were identified as small, round, well-defined, hypointense lesions on SWI, 2–10 mm in size, and that are not well seen on a T2 sequence. They were distinguished from microbleed mimics such as signals from air-bone interfaces, mineralization in globi pallidi or dentate nuclei, hemorrhage from a specific secondary cause, and partial volume artifacts using Microbleed Anatomic Rating Scale criteria [22]. The number of CMB and the coexistence of cortical superficial siderosis were analyzed. The CMB counts obtained from the follow-up MRI scans were compared to the baseline CMB counts. Individuals with elevated CMB counts at follow-up were classified as having CMB progression. We compared individuals with and without CMB progression to identify potential associations between demographic and clinical risk factors and CMB development.

### 4.4. Computed Tomography (CT) Interpretation and Cerebral Arterial Calcification Score

The cerebral arterial calcification score was obtained by conducting CT imaging. For analysis of the CT images, all raw Digital Imaging and Communications in Medicine (DICOM) files from the axial reconstruction were downloaded, anonymized, and converted to NifTi format. For each CT volume, a threshold-based technique was used to manually generate regions of interest for calcification within the intracranial vasculature using the open-source 3D Slicer platform version 4.8 [23]. Vessels of interest were the internal carotid, external carotid, and vertebral arteries as well as the basilar artery. A custom Python script was written to determine the total number of voxels containing calcification and converted to physical volume (cm^3^) through multiplication by voxel size contained in the original DICOM header. All masks were visually inspected for accuracy by a board-certified subspecialist neuroradiologist. Total calcification scores were calculated by summing up the calcification physical volumes in all arterial regions of interest per human brain. The subjects who underwent CT imaging were stratified into tertiles based on their total calcification scores. 

### 4.5. Clinical Outcomes

The primary outcome was the occurrence of ischemic or hemorrhagic stroke after the initial evaluation of patients with CMB. Clinical stroke symptoms reported by the patients were compared with radiologic abnormalities on MRI studies obtained at follow-up. MRI studies were used to characterize the stroke as ischemic or hemorrhagic and the likely underlying mechanism. Our secondary outcome was the progression of CMB, defined as an increase in the number of CMB detected on the follow-up MRI compared with the initial MRI.

### 4.6. Statistical Analysis

Descriptive statistics (means, standard deviations, proportions) were calculated for the overall sample and stratified by the progression of CMB. For calculation of stroke incidence rates, the person-years of follow-up for each participant was calculated from the initial visit to the follow-up visit or the time of stroke, whichever came first. Group differences in the means of continuous variables and in proportions of categorical variables were tested using t-tests and Fisher exact tests. Two-sided *p* < 0.05 was considered statistically significant. Statistical analyses were performed using SAS software version 9.4 for Windows (SAS Institute Inc., Cary, NC, USA).

## 5. Conclusions

In this stroke clinic-based study, we demonstrated that CMB presence was associated with an overall benign clinical course. In addition, patients with higher cerebral arterial calcification scores had a significantly greater CMB burden. Prospective longitudinal studies and pathology analyses are needed to obtain a better understanding of the relationship between vascular calcification and CMB formation, and to determine whether cerebral arterial calcification score may be useful in cerebral microbleed risk prediction.

## Figures and Tables

**Table 1:** Demographic factors and stroke events in 46 patients with and without cerebral microbleed (CMB) progression.

		CMB No Progression (*n* = 21)	CMB Progression (*n* = 25)	*p*-Value
		Mean ± SD	Mean ± SD	
Age		73.3 ± 12.1	73.7 ± 10.4	0.90
Clinical follow-up interval (months)		24.2 ± 23.3	29.1 ± 22.1	0.46
MRI follow-up interval (months)		18.6 ± 12.1	33.1 ± 18.8	0.003
		N (%)	N (%)	
Male		12 (57)	14 (56)	1.00
Race	White	6 (29)	14 (56)	0.08 (White vs. Other)
	Asian	8 (38)	9 (36)	
	Hispanic	5 (24)	2 (8)	
	Black	2 (10)	0 (0)	
Hypertension		14 (67)	22 (88)	0.15
Diabetes		5 (24)	7 (28)	1.00
Hyperlipidemia		13 (62)	15 (60)	1.00
Smoking		3 (14)	4 (16)	1.00
Chronic kidney disease		5 (24)	8 (32)	0.22
Cerebral amyloid angiopathy		3 (14)	6 (24)	0.48
Use of antithrombotics		13 (62)	15 (60)	1.00
Stroke event at follow-up		1 (5)	3 (12)	0.61

**Table 2:** Vascular calcification scores and cerebral microbleed (CMB) counts in 26 patients.

Calcification Score (cm^3^)	Number of CMB at Initial Scan	Number of CMB at Follow-Up Scan
	Mean ± SD Range No. of Subjects	Mean ± SD Range No. of Subjects
<300 Range 32–286	4.9 ± 2.2 1–7 (*n* = 8)	5.6 ± 2.4 3–8 (*n* = 5)
300–1000 Range 309–770	12.3. ± 12.4 0–32 (*n* = 9) †	13.7 ± 13.5 0–32 (*n* = 9) †
>1000 Range 1159–4021	13.0 ± 11.7 3–37 (*n* = 8)	15.0 ± 8.6 7–27 (*n* = 4)
*p*-value for difference in low vs. intermediate and high groups	0.02	0.03

† One patient with many but not quantifiable CMB on both initial and follow-up MRI was not included in the calculation.
